# Dietary intakes in infertile women a pilot study

**DOI:** 10.1186/1475-2891-8-53

**Published:** 2009-11-10

**Authors:** Ottavia Colombo, Giovanna Pinelli, Mario Comelli, Pierpaolo Marchetti, Sabina Sieri, Furio Brighenti, Rossella E Nappi, Anna Tagliabue

**Affiliations:** 1 Department of Applied Health Sciences, University of Pavia, via Bassi 21, 27100 Pavia, Italy; 2 Nutritional Epidemiology Unit, National Cancer Institute, Via Venezian 1, 20133 Milan, Italy; 3 Department of Public Health, University of Parma, via Volturno 39, 43100 Parma, Italy; 4 Research Centre for Reproductive Medicine, Dept of Morphological, Eidological and Clinical Sciences & Gynecological Endocrinology Unit, Dept of Internal Medicine & Endocrinology, IRCCS S. Maugeri Foundation, University of Pavia, Via Ferrata 8 - 27100 Pavia, Italy

## Abstract

**Background:**

The reproductive axis is closely linked to nutritional status. The purpose of this study was to compare the nutritional status in two groups of young infertile women, without clinically overt eating disorders: hypothalamic amenorrhea (HA) and polycystic ovary syndrome (PCOS).

**Methods:**

Eighteen young infertile women (10 HA, 8 PCOS) attending an outpatient gynecological endocrinology unit, underwent evaluation of anthropometry, body composition, dietary intakes by means of a food frequency questionnaire (FFQ) and a seven-day food diary (7DD), and psychological characteristics by means of EDI2 and SCL90 tests.

**Results:**

HA women had lower BMI and body fat compared to PCOS women. Habitual intake derived from FFQs showed a similar macronutrient distribution between groups (about 16% protein, 33% fat, 52% carbohydrates). The psychometric profiles of the two groups did not differ significantly. The underreporting of dietary intakes (measured as habitual energy intake by FFQs/basal metabolic rate) was found to be negatively correlated with the interpersonal sensitivity SCL-90 subscale scores (r = -0.54, p = 0.02).

**Conclusion:**

Our study identified differences in body composition but not in dietary habits between HA and PCOS infertile women. We documented, for the first time, a relationship between the accuracy of dietary surveys and the psychological characteristics of subjects with anovulation. This finding suggests that it may be important to be aware of the psychological terrain when planning a dietary survey in infertile women.

## Background

Adequate nutritional status is a critical determinant of the onset and maintenance of normal reproductive function [1]. In 1974, Frisch and McArthur already wrote that weight loss causes loss of menstrual function (amenorrhea) and weight gain restores menstrual cycles [2]. Further studies showed that energy balance is more important than body fat mass itself for ovulatory function, since in some cases recovery may occur after minimal reacquisition of weight, or even long before there is any change in body weight or an increase in body fat [3].

Hypotalamic amenorrhea (HA) and polycystic ovary syndrome (PCOS) represent two common causes of infertility [4,5] in which alterations of nutritional status have been documented [6-13].

Nutritional deficits may represent a common contributing factor in the development and maintenance of multiple neuroendocrine-metabolic aberrations underlying functional HA [6,7]. Few studies investigated in detail dietary habits and body composition in HA. A reduced body fat percentage and low fat intake is documented by Laughlin [6] and by Cousinet [8] in two groups of HA.

Women suffering from PCOS have been shown to have higher amount of body fat compared to healthy controls even when they are normal weight [9]. As far as dietary habits are concerned, Douglas and colleagues did not find any association between dietary variables and metabolic abnormalities in PCOS, but they reported a trend in higher consumption in total fat, saturated fat and total servings of high glycemic index foods [10]. Carmina compared dietary intakes in two groups of American and Italian PCOS women and found that the amount of saturated fat consumed by American women was almost double than that in Italian ones and correlated significantly with HDL-C [11]. An increased risk of ovulatory infertility has been associated to higher consumption in low fat dairy foods and in glycemic load in recent longitudinal studies by Chavarro and colleagues [12,13].

An improvement in dietary habits in order to reach an adequate nutritional status can be needed both in HA and in PCOS, consequently it would be recommended to investigate dietary intakes in infertile women (and to compare them to nutritional recommendations) in order to evaluate the possibility to correct them.

Therefore, the purpose of our study was to assess the nutritional status in young infertile women without clinically overt eating disorders, comparing dietary habits and body composition in two different types of infertility: PCOS and hypothalamic amenorrhea.

Given the evidence that physical and psychological characteristics of study participants play an important role in the observed reporting bias of dietary intakes [14] and that the loss of menstrual function represents a notable psychological distress for infertile women [15,16] we included their psychological profile in the assessment.

## Materials and methods

### Subjects

Outpatients attending a gynecological endocrinology unit were enrolled in the present study, after they have given informed consent. They were selected according to the following criteria: age 18-35 years; menstrual dysfunction and infertility; stable weight in the past three months. The exclusion criteria were: hyperprolactinemia; thyroid pathologies or other endocrine disorders; clinically overt eating disorders, past or present use of psychoactive agents. The study sample recruited comprised 18 young women with anovulation: 10 met the diagnostic criteria for functional hypothalamic amenorrhea (group A) [4] and 8 for polycystic ovary syndrome (group B) [5]. All the subjects gave their informed consent to participate in the study as described below. The study was approved by the ethics committee of the University of Pavia.

### Procedure

Each patient underwent a complete medical history and a psycho-physical assessment, including blood chemistry for hormone concentration, anthropometry and body composition analysis, evaluated by dual-energy x-ray absorptiometry (DXA), dietetic assessment, and psychometric tests. For each patient all these procedures required 3 visits, each of them lasting about 90 min. The subjects were instructed not to change their usual eating habits throughout the duration of the study and dietary intakes were assessed on the basis of the data collected using two instruments: a self-administered food frequency questionnaire (FFQ) and a seven-day food diary (7DD); for each subject recorded data were then compared to Dietary Reference Intakes [17,18]. In addition, a trained dietician assessed the glycemic index and glycemic load of each patient's diet, using a computerized system.

### Anthropometry

Body height was measured to the nearest 0.5 cm using a wall-mounted stadiometer and weight (in light underwear) to the nearest 0.1 kg using a balance beam scale. Body mass index (BMI) was calculated in the standard way: weight in kg divided by height in m^2^. With the patients standing, their waist circumference was measured to the nearest 1 mm with a measuring tape placed at the midpoint between the lower border of the ribs and the upper border of the pelvis.

### Body composition

Body composition was assessed by dual x-ray energy absorptiometry (DXA) using a Norland RX-26 scanner (Norland Corp., W, USA). DXA measures total body bone mineral content (BMC) and density (BMD), and fat and lean mass.

The scanner uses an x-ray source; the extinction of x-rays, which is dependent on the tissue, is measured and absolute and relative fat mass and lean body mass are estimated. Total body scans were performed with subjects in the supine position. The entire body of each subject was scanned, beginning at the top of the head, with the "medium t" scan mode. The mean measurement time was 15 min; radiation exposure was < 7 Sv. Daily quality-assurance tests were performed according to the manufacturer's directions. All scans were performed and analysed by the same operator.

### Hormone concentration

The examination started with the quantitative determination of hormone levels, in order to meet the criteria for the diagnosis of HA or PCOS. The following hormones were determined: 17βestradiol, follicle-stimulating hormone (FSH), luteinizing hormone (LH), prolactin, progesterone, testosterone, dehydroepiandrostendionsulfate (DHEA-S), androstenedione, cortisol. Thyroid function was also evaluated. Blood samples were collected between 7.30 and 9.30 am and hormones were measured by commercially available kits at the Research Centre for Reproductive Medicine.

### Seven-day food diary

In order to assess recent dietary habits, participants were asked to take home and complete the 7DD in the standard way [19,20]. The diary contains instructions and pages for recording foods eaten at six meals (breakfast, mid-morning snack, lunch, afternoon snack, dinner, after-dinner snack) on each of the seven days of the week. It is clearly explained in the instructions that respondents should record both the brands of food eaten and the size of the portions consumed each day. A trained dietitian checked the 7 day diary for completeness according to a standardized procedure [20]. Energy and nutrient intake analysis was carried out using a computerized system (Dieta ragionata 7.0 - ESI informatica, San Donato Milanese - Milan, 1997) developed using published Italian food composition tables [21,22].

### Food frequency questionnaire

In order to assess past dietary habits, the FFQ, which is used to assess long-term food intake, was developed and validated [23,24] in the framework of the European Prospective Investigation into Cancer and Nutrition (the EPIC study). The instrument, which is designed to ascertain in detail how much and what kinds of food were consumed during the previous year, contains 254 questions about 188 different food items; it gives illustrations of three sample sizes of dish or references to standard portion sizes. The food categories investigated are cereals, vegetables, fruit, meat, fish, dairy products, sweet foods, alcoholic and non alcoholic beverages. Questions on seasoning and food preparation are also included.

Participants could specify the frequency of consumption of items by day, week, month or year. The average daily nutrient intake was calculated by multiplying the frequency of consumption of each food portion by its nutrient content, as set out in the Italian food composition tables [22]. The FFQ was self-administered; the completed questionnaire was reviewed by a trained member of staff together with the participant to fill in any missing items.

### Glycemic index and glycemic load

Pre-coded forms were used to investigate consumption of carbohydrate-rich foods, such as bread, sweet foods and carrots, and establish the glycemic index and the glycemic load of the patient's diet. The Department of Public Health of the University of Parma created the computerized system used in this part of the investigation and also evaluated the results [25].

### Validity of dietary assessment methods and comparison of results with DRI

The ratio of reported energy intake to basal metabolic rate (BMR) calculated according to Schofield [26] (validity index) was used to identify misreporting [14]. Individuals whose reported energy intake was less than 1.2 times their BMR were defined Low Energy Reporters (LERs) [27]. This cutoff was chosen on the basis of WHO/FAO estimations of the lowest plausible energy intakes at weight maintenance [14,28].

The adequacy of macro and micronutrient intakes was evaluated by using the Estimated Average Requirement (EAR) cut-point method (calculating the proportion of individuals in the group with intakes below the EAR) [18].

### Psychometric tests

Each subject filled in the following questionnaires: the Eating Disorder Inventory (EDI2) and the Symptom Check List-90 (SCL-90). The SCL-90 is a 90-item tool designed to identify psychological distress. For each item, patients are required to rate how distressing they found the given problem during the previous week, on a scale of 0 (not at all) to 4 (extremely). The items are divided into nine domains (somatization, obsessive-compulsive, interpersonal sensitivity, depression, anxiety, hostility, phobic anxiety, paranoid ideation, and psychoticism); there is also a global severity index (GSI), which is used as an indicator of overall psychological distress [29]. The EDI2 is a 91-item questionnaire with 11 subscales designed to quantify behavioral and cognitive features of anorexia nervosa and bulimia (drive for thinness, bulimia, body dissatisfaction, ineffectiveness, perfectionism, interpersonal distrust, interoceptive awareness, maturity fears, ascetism, impulse regulation, social insecurity) [30]. For the analysis of the data, we referred to the Italian adaptation of the questionnaire, which contains data for the Italian population [31].

### Statistical analysis

Descriptive statistics were preliminary calculated for all the variables divided into two groups according to the gynecological diagnosis. All variables distribution was tested with Kolmogorov-Smirnov test which indicated that normal distribution could be assumed for all of them. Student t-test was used to assess between-groups differences for anthropometric parameters, dietary intakes and psychological scores. Differences between the psychometric scores of the study population and normative data from the literature were tested using the t test with the Welch correction for unequal variances. Correlations among the variables were examined using Pearson product-moment correlations. For descriptive purposes mean values ± standard deviations are reported. A level of p < 0.05 was accepted as statistically significant. All analyses were performed using the SPSS/PC software program (version 15.0, SPSS Inc, Chicago).

## Results

### Characteristics of the subjects

Participants were Caucasian women living in Pavia. The majority of women in both groups had a higher education (60.0% in HA and 62.5% in PCOS) and had never been married (90% in HA and 100% in PCOS). In HA group 60% of women had a job, while 40% were student; in PCOS group 50% of women were student, 25% had a job, and 25% were unemployed. None of them was engaged in sport activities in their leisure time.

### Anthropometric and metabolic characteristics of the subjects

Table 1 shows the anthropometric and metabolic characteristics of the study population.

**Table 1:** Anthropometry, body composition and metabolic characteristics of the two groups of infertile women.

Variables	GROUP A^1^ n = 10 (mean ± sd)	GROUP B^2^ n = 8 (mean ± sd)	p-value
Age (years)	26.0 ± 4.4	21.0 ± 3.2	*0.016*
Weight (kg)	54.1 ± 6.9	67.2 ± 7.9	*0.002*
Height (cm)	164.9 ± 3.1	166.9 ± 9.7	*0.594*
BMI (kg/m^2^)	19.9 ± 2.3	24.3 ± 3.5	*0.006*
WC(cm)	69.6 ± 4.5	81.3 ± 6.8	*<0.001*
Body fat (kg)	13.9 ± 5.8	23.8 ± 8.8	*0.014*
Body fat (% weight)	25.3 ± 7.5	35.2 ± 9.4	*0.030*
Lean body mass (kg)	37.9 ± 4.1	40.1 ± 2.8	*0.211*
BMR (kcal/24 h)	1287.2 ± 96.4	1483.4 ± 116.9	*0.001*

BMI = body mass index; WC = waist circumference; BMR = basal metabolic rate

^1 ^hypothalamic amenorrhea; ^2 ^polycystic ovary syndrome

The 18 infertile women had a mean age of 24 years (range 18-33 years). The patients in group A (HA) were significantly older and had significantly lower body weight, BMI, waist circumference, body fat mass and body fat percentage, and BMR than the women in group B (PCOS) (p < 0.05).

### Dietary intakes

The energy intakes calculated from the data collected using each of the two tools were not correlated with each other (r = 0.321, p = 0.2) and values derived from the 7DDs were significantly lower than those derived from the FFQs (Table 2). Considering data reported in the 7DDs, 12 women (67%) reported energy intakes less than 1.2 times their BMR and were thus classified as LERs (6 in group A and 6 in group B). Therefore, results obtained from the 7DDs were excluded from the subsequent analyses due to the high percentage of LERs observed.

**Table 2:** Energy intakes calculated from 7-day diaries and food frequency questionnaires in the two groups of infertile women.

Variables	GROUP A^1^ n = 10 (mean ± sd)	GROUP B^2^ n = 8 (mean ± sd)
*7-day food diary*		
Energy (kcal/24 h)	1396.1 ± 435.1	1545.4 ± 432.9
Energy intake/BMR	1.10 ± 0.36	1.04 ± 0.29
LERs (% subjects)	60.0	75.0
		
*Food frequency questionnaire*		
Energy (kcal/24 h)	2010.1 ± 492.1 ^a^	2471.2 ± 676.1 ^a^
Energy intake/BMR	1.58 ± 0.44 ^b^	1.67 ± 0.46 ^b^
LERs (% subjects)	20.0	12.5

Energy intake/BMR: ratio of reported energy intake to basal metabolic rate; LERs = low energy reporters

^1 ^hypothalamic amenorrhea; ^2 ^polycystic ovary syndrome

^a ^p < 0.05 between the mean energy intakes BMR calculated from the two instruments (7-day food diary *vs*. food frequency questionnaire)

^b ^p < 0.05 between the energy intake/BMR calculated from the two instruments (7-day food diary *vs*. food frequency questionnaire)

Considering data reported in the FFQs, 3 women (17%) reported energy intakes less than 1.2 times their BMR with this method and were thus classified as LERs (2 in group A and 1 in group B). No differences emerged between the two groups as regards the mean calculated energy intake/BMR ratio (group A 1.58 ± 0.44, group B 1.67 ± 0.46, p = 0.67).

The habitual intake of energy and macronutrients, calculated from the FFQs, is shown in Table 3. Energy intake as well as protein, carbohydrate and lipid intakes did not differ significantly between group A and group B, neither in absolute values nor when expressed per kg of lean body mass; fat from animal origin and saturated fat intakes were significantly lower in group A than in group B (p < 0.05), but the difference was no more significant when adjusted per kg of lean body mass. The glycemic load and glycemic index did not differ significantly between the two groups. As regards the percentage distribution in macronutrients, it was similar between groups (about 16% protein, 33% fat, 52% carbohydrates), and fell within the recommended ranges [17] for the majority of subjects in both groups.

**Table 3:** Daily dietary intakes of macronutrients in infertile women assessed using the food frequency questionnaire (FFQ).

Variables		Dietary Reference Intakes (DRIs)	GROUP A^1 ^n = 10 (mean ± sd)	GROUP B^2 ^n = 8 (mean ± sd)
**Energy Intake**				
Energy (kcal/d)			2010.1 ± 492.1	2471.2 ± 676.1
Energy intake/BMR			1.58 ± 0.44	1.67 ± 0.46
Energy (kcal/FFMkg/d)			54.3 ± 16.4	62.5 ± 19.8

**Daily Intakes (% energy)**				
Protein		10-35 ^a^	16.7 ± 3.9	16.1 ± 2.3
Fat		20-35^a^	32.0 ± 9.1	33.8 ± 3.7
Carbohydrate		45-65 ^a^	50.6 ± 7.4	53.0 ± 5.0

**Daily Intakes (g/d)**				
Protein		38^b^	82.9 ± 21.7	96.7 ± 19.4
Fat		ND^b^	71.7 ± 26.1	91.7 ± 22.5
	Animal fat		32.0 ± 18.4	49.3 ± 15.5 *
	Saturated fat		21.4 ± 9.9	30.8 ± 8.9 *
	Monounsaturated fat		34.9 ± 13.4	43.6 ± 10.7
	Polyunsaturated fat		11.0 ± 7.7	12.0 ± 3.6
	Cholesterol (mg/d)		291.6 ± 115.1	347.5 ± 108.7
Carbohydrates		100 ^b^	256.6 ± 82.9	331.4 ± 116.6
	Starch		130.4 ± 61.1	168.2 ± 64.9
	Soluble sugars		125.7 ± 56.4	162.7 ± 63.2
Fiber		25 ^b^	24.3 ± 5.1	24.4 ± 8.1
Glycemic load			107.4 ± 45.9	128.8 ± 57.0
Glycemic index			52.2 ± 2.7	54.5 ± 3.8
Water (ml/d)		2700 ^c^	1675.7 ± 690.0	1606.8 ± 698.5
Alcohol			9.5 ± 15.7	1.6 ± 1.6

**Daily Intakes (g/FFMkg/d)**				
Protein			2.2 ± 0.7	2.4 ± 0.6
Fat			1.9 ± 0.8	2.3 ± 0.7
	Animal fat		0.9 ± 0.5	1.3 ± 0.5
	Saturated fat		0.6 ± 0.3	0.8 ± 0.3
	Monounsaturated fat		0.9 ± 0.4	1.1 ± 0.3
	Polyunsaturated fat		0.3 ± 0.2	0.3 ± 0.1
	Cholesterol (mg/FFMkg/d)		7.9 ± 3.4	8.9 ± 3.3
Carbohydrates			6.9 ± 2.6	8.4 ± 3.2
	Starch		3.6 ± 1.9	4.2 ± 1.7
	Soluble sugars		3.4 ± 1.6	4.1 ± 1.7

Energy intake/BMR: ratio of reported energy intake to basal metabolic rate

^1 ^hypothalamic amenorrhea; ^2 ^polycystic ovary syndrome

^a^Acceptable Macronutrients Distribution Ranges

^b^Estimated Average Requirements for normal-weight women, age range 19-30

^c ^Adequate Intakes for normal-weight women, age range 19-30

* p < 0.05, mean values were significantly different between group A and group B

According to the Estimated Average Requirement (EAR) cut-point method (calculating the proportion of individuals in the group with intakes below the EAR) [18] it was possible to estimate that all subjects had an appropriate intake in protein and carbohydrates, while a high percentage of subjects in both group had an inappropriately low intake in fiber (60% in group A and 50% in group B, respectively) and water (90% in group A and 87.5% in group B, respectively).

A detailed description of micronutrient intakes has been reported elsewhere (manuscript in preparation).

### Psychometric assessment

**Figure 1:**
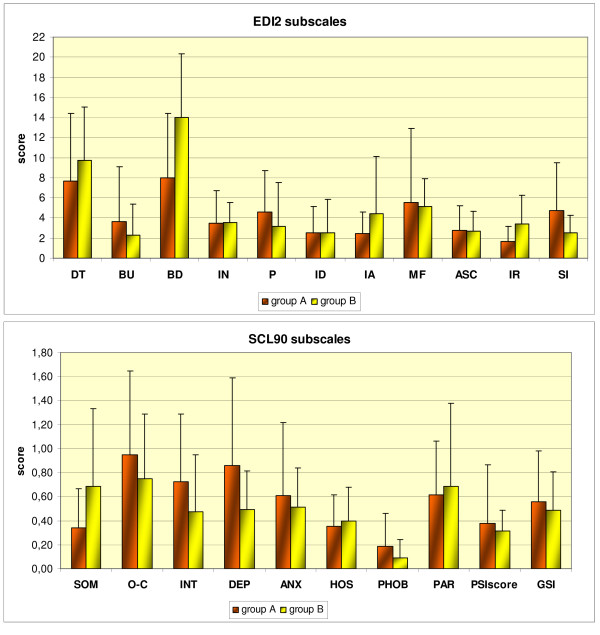
**Psychometric profiles of infertile women with hypothalamic amenorrhea (group A) and polycystic ovary syndrome (group B)**. Scores are expressed by mean value and standard deviations. *Upper panel: *EDI-2 subscales are indicated as follows: DT: drive for thinness, BU: bulimia, BD: body dissatisfaction, IN: ineffectiveness, P: perfectionism, ID: interpersonal distrust, IA: interoceptive awareness, MF: maturity fears, ASC: ascetism, IR: impulse regulation, SI: social insecurity. *Lower panel: *SCL90 subscales are indicated as follows: SOM: somatization, O-C: obsessive-compulsive, INT: interpersonal sensitivity, DEP: depression, ANX: anxiety, HOS: hostility, PHOB: phobic anxiety, PAR: paranoid ideation, PSY: psychoticism, GSI: global severity index.

The psychometric profiles of the two groups are illustrated in Figure 1.

No significant difference emerged between Group A and Group B either in total EDI2 scores (group A: 47.1 ± 21.3 vs. group B: 53.1 ± 24.1, p = 0.6) or in any of the EDI2 subscale scores. The mean values were comparable with normative data from age-matched healthy women [31], except for some subscales, on which infertile women recorded scores lower than control values: interoceptive awareness, ascetism and impulse regulation in group A (2.4 ± 2.1 vs. 5.2 ± 4.8, p < 0.005; 2.8 ± 2.4 vs. 5.1 ± 3.3, p = 0.02; and 1.7 ± 1.5 vs. 5.2 ± 5.1, p < 0.001, respectively), and ascetism and social insecurity in group B (2.7 ± 2.0 vs. 5.1 ± 3.3, p = 0.02, and 2.6 ± 1.7 vs. 4.5 ± 3.6, p = 0.02, respectively). Moreover, although the mean values were comparable with normative data from age-matched healthy women, 6 of the 18 subjects (33%) (3 in group A) gave high scores on the drive for thinness subscale, and 4 of the 18 (22%) (2 in group A) gave high scores on the body dissatisfaction subscale.

With regard to the SCL-90, no significant differences were found between the groups either in the GSI (group A: 0.56 ± 0.42 vs. group B: 0.49 ± 0.32, p = 0.58) or in any of the subscale scores. Furthermore, all the results were comparable with normative data from age-matched healthy women [32] except for the phobic anxiety subscale, on which women affected by PCOS recorded scores lower than control values (0.09 ± 0.15 vs. 0.28 ± 0.40, p = 0.02).

The validity index (reported energy intake/BMR ratio) derived from the FFQs showed some correlations with psychometric variables. If we consider the entire group of infertile women taken together, the validity index was found to be negatively correlated with the interpersonal sensitivity (r = -0.54, p = 0.02) SCL-90 subscale scores. Considering the HA group alone, the validity index was found to be negatively correlated with the interpersonal sensitivity (r = -0.66, p = 0.038) and somatization (r = -0.68, p = 0.032) SCL-90 subscale scores. No correlations were found in the PCOS group alone.

## Discussion

The present study investigated the nutritional status and dietary habits of infertile women, comparing values from women affected by hypothalamic amenorrhea to those of PCOS women.

Women affected by PCOS were found to have higher BMI than women affected by functional HA, nevertheless their mean BMI fell within the range of normal weight according to WHO cut-offs [33], thus confirming that a condition of overweight or obesity is frequent but not constant in PCOS, being observed in about 20% of affected subjects in Italy (unpublished observation). Besides, PCOS women showed a significantly different body composition and distribution: their percentage body fat resulted higher both than HA and than reference values in normal-weight women of the same age [34]. This is in agreement with previous studies, showing that an increased body fat is present not only in the majority of women with PCOS who are obese, but also occurs in overweight and normal-weight women affected by the syndrome [9,35].

As regards dietary habits, our study revealed an adequate energy intake in both groups of women and the macronutrient distribution found fell within the distribution ranges recommended for the healthy population [17]. Considering absolute values, women with PCOS consumed more fat from animal origin and more saturated fat, confirming the high consumption of saturated fat previously observed in American [10] and Italian women [11] with PCOS. In our study groups the greater amount of dietary fat and saturated fat could be explained by the higher energy intake, and the difference disappeared when dietary intakes were normalized for body composition. The cross sectional and exploratory nature of this study does not allow us to investigate the relationship between fat consumption and body composition in PCOS women, for which future prospective studies on larger populations are needed.

The amount and quality of carbohydrates in diet may be important determinants of ovulation and fertility in healthy women, as suggested by recent studies [10,12,13], therefore we included in our study the evaluation of glycemic index and glycemic load, which did not show any significant difference between the two groups.

In our population, an excessively high percentage of Low Energy Reporters was found with the 7DD method of data collection, leading to the exclusion of 7DD-derived data from the subsequent analyses. On the contrary, the percentage of LERs observed with the FFQ was lower than that reported in one other study comparing 7DD and FFQ [36] and the mean ratio of reported energy intake to BMR, about 1.6, is compatible with an active lifestyle and a stable weight, as observed in our population of young women. The 7DD was found to be superior to the FFQ when assessed in comparison with weighed records and biomarkers [37], but it demands more time and attention because it has to be filled in after every meal or snack, and is therefore less readily accepted by some patients. This is the reason why also several other studies developed different methods, other than dietary diary, to assess dietary intakes [10-13].

Dietary intakes and reporting are indeed influenced by psychological correlates and life events, but these parameters are not usually included in dietary surveys. In this work we studied psychological profiles by means of two validated psychometric tests and observed some significant relationships between subscale scores and dietary intakes measured using the FFQ. The significant negative correlation between the energy intake/BMR ratio and the interpersonal sensitivity subscale of SCL90 was particularly interesting and, to our knowledge, has never been reported in previous studies. This subscale focuses on feelings of personal inadequacy and inferiority, particularly in comparison to other individuals. Self-deprecation, feelings of uneasiness, and marked discomfort during interpersonal interactions are characteristic manifestations of this psychological dimension, as are acute self-consciousness and negative expectancies regarding interpersonal communications.

Underreporting is usually due to subjects modifying their food choices or underreporting their actual food intake. The above-mentioned negative correlation emerging in our study indicates that people who are more prone to feelings of personal inadequacy and inferiority will show an increased tendency to underreport, which leads to a lower ratio between reported energy intake and BMR (validity index).

The study has some limitations. First, the small number of subjects in each study group does not permit to draw definitive conclusions. Both HA and PCOS are heterogeneous disorders and our exploratory findings need to be confirmed in larger samples.

Second, the adequacy of dietary assessment could be improved by adding a measure of energy expenditure by use of indirect calorimetry for the measurement of BMR or, whenever possible, the use of Doubly Labeled Water for the measurements of 24 h energy expenditure.

Lastly, the cross sectional nature of the study does not permit to establish a relationship between observed dietary intakes and body composition of our subjects.

## Conclusion

Our study identified differences in body composition but not in dietary habits (except for animal and saturated fat) between infertile women affected by hypothalamic amenorrhea and PCOS women. Moreover we documented, for the first time, a relationship between the accuracy of dietary surveys and the psychological characteristics of subjects with anovulation. This finding suggests that it may be important to be aware of the psychological terrain when planning a dietary survey in infertile women.

## Competing interests

The authors declare that they have no competing interests.

## Authors' contributions

OC: conception and design of the study; acquisition, analysis and interpretation of data; drafting of the manuscript; approval of the final version of the manuscript. GP: conception and design of the study; acquisition, analysis and interpretation of data; drafting of the manuscript; approval of the final version of the manuscript. MC: statistical analysis and interpretation of data; approval of the final version of the manuscript. PM: statistical analysis and interpretation of data; approval of the final version of the manuscript. SS: analysis of dietary intakes and interpretation of data; approval of the final version of the manuscript. FB: analysis of dietary intakes and interpretation of data; approval of the final version of the manuscript. REN: conception and design of the study; recruitment of patients; interpretation of data; approval of the final version of the manuscript. AT: conception and design of the study; acquisition, analysis and interpretation of data; drafting of the manuscript; approval of the final version of the manuscript. All the authors have read and approved the final manuscript.
